# First Colombian Multicentric Newborn Screening for Congenital Toxoplasmosis

**DOI:** 10.1371/journal.pntd.0001195

**Published:** 2011-05-31

**Authors:** Jorge Enrique Gómez-Marin, Alejandra de-la-Torre, Edith Angel-Muller, Jorge Rubio, Jaime Arenas, Elkin Osorio, Lilian Nuñez, Lyda Pinzon, Luis Carlos Mendez-Cordoba, Agustin Bustos, Isabel de-la-Hoz, Pedro Silva, Monica Beltran, Leonor Chacon, Martha Marrugo, Cristina Manjarres, Hernando Baquero, Fabiana Lora, Elizabeth Torres, Oscar Elias Zuluaga, Monica Estrada, Lacides Moscote, Myriam Teresa Silva, Raul Rivera, Angie Molina, Shirley Najera, Antonio Sanabria, Maria Luisa Ramirez, Claudia Alarcon, Natalia Restrepo, Alejandra Falla, Tailandia Rodriguez, Giovanny Castaño

**Affiliations:** 1 Grupo GEPAMOL, Centro de Investigaciones Biomédicas, Universidad del Quindio, Armenia, Colombia; 2 Facultad de Medicina, Universidad Nacional de Colombia, Bogota, Colombia; 3 Laboratorio de Salud Pública, Secretaría Distrital de Salud, Bogotá, Colombia; 4 Instituto Materno Infantil, Bogota, Colombia; 5 Hospital de Engativa, Bogota, Colombia; 6 Clínica Medilaser, Florencia, Colombia; 7 Universidad de Santander, Bucaramanga, Colombia; 8 Universidad Autónoma de Bucaramanga, Bucaramanga, Colombia; 9 Laboratorio Salud Pública de Santander, Bucaramanga, Colombia; 10 Universidad del Norte, Barranquilla, Colombia; 11 Hospital Universitario San Juan de Dios de Armenia, Armenia, Colombia; 12 Hospital Nuestra Señora de los Remedios, Riohacha, Colombia; 13 Hospital Erasmo Meoz, Cúcuta, Colombia; 14 Clinica Colombia, Colsanitas, Bogotá, Colombia; 15 Hospital Simón Bolívar, Bogotá, Colombia; 16 Universidad Javeriana, Bogota, Colombia; Yale Child Health Research Center, United States of America

## Abstract

**Aims:**

To determine the incidence of congenital toxoplasmosis in Colombian newborns from 19 hospital or maternal child health services from seven different cities of five natural geographic regions (Caribbean, Central, Andean, Amazonia and Eastern).

**Materials and Methods:**

We collected 15,333 samples from umbilical cord blood between the period of March 2009 to May 2010 in 19 different hospitals and maternal-child health services from seven different cities. We applied an IgM ELISA assay (Vircell, Spain) to determine the frequency of IgM anti *Toxoplasma*. The results in blood cord samples were confirmed either by western blot and repeated ELISA IgM assay. In a sub-sample of 1,613 children that were negative by the anti-*Toxoplasma* IgM assay, the frequency of specific anti-*Toxoplasma* IgA by the ISAGA assay was determined. All children with positive samples by IgM, IgA, clinical diagnosis or treatment during pregnancy were recalled for confirmatory tests after day 10 of life.

**Results:**

61 positive samples for specific IgM (0.39%) and 9 positives for IgA (0.5%) were found. 143 questionnaires were positive for a clinical diagnosis or treatment for toxoplasmosis during pregnancy. 109 out of the 218 children that had some of the criteria for postnatal confirmatory tests were followed. Congenital toxoplasmosis infection was confirmed in 15 children: 7 were symptomatic, and three of them died before the first month of life (20% of lethality). A significant correlation was found between a high incidence of markers for congenital toxoplasmosis and higher mean annual rainfall for the city.

**Conclusions:**

Incidence for congenital toxoplasmosis is significantly different between hospitals or maternal child health services from different cities in Colombia. Mean annual rainfall was correlated with incidence of congenital toxoplasmosis.

## Author Summary

Congenital toxoplasmosis can result in permanent sequel as blindness or neurological damage in children and it seems to be more severe in South America than in other continents. There is a lack of information about this frequency in Colombia, where no control program is established, although it is a recognized cause of potentially preventable congenital blindness. We propose the first Colombian multicentric study to determine the frequency and impact of congenital toxoplasmosis. More than 15,000 newborns in seven cities were studied. Newborns were tested at birth by doing a cord blood test for toxoplasmosis. Additionally, children from mothers with history of toxoplasmosis acquired during pregnancy were recalled for a follow-up. The program identified fifteen children otherwise undiagnosed; three of these children died as consequence of congenital toxoplasmosis. The frequency of the congenital infection varied significantly between cities, being higher in Armenia and Florencia, intermediate in Bogota, Bucaramanga and Barranquilla and very low in western cities such as Cucuta and Riohacha. For the first time a significant correlation was found between mean rainfall at the city and the incidence of this congenital infection.

## Introduction

Congenital toxoplasmosis is generally the result of a primary infection during pregnancy. The clinical manifestation of the infant will depend of the gestational week when the mother acquired the infection and is characterized by a broad spectrum of symptoms at birth, including varying degrees of neurologic, ophthalmologic and systemic involvement [1]. Recent reports indicate that congenital toxoplasmosis is more often symptomatic in South America than in Europe. This was demonstrated when cohorts of congenitally infected children from different continents were compared [2]. The greater severity of South American cases was an unexpected result of the SYROCOT international collaborative study [2]. Additionally, a comparative prospective cohort study of congenitally infected children in Brazil and Europe found that Brazilian children had eye lesions that were larger, more numerous, and more likely to affect the part of the retina responsible for central vision, compared with their counterparts in Europe [3]. The authors of the study suggested that the increased frequency and severity of ocular disease in Brazil compared with Europe was due to exposure to more virulent strains of *Toxoplasma gondii* in Brazil [3]. Importantly, the parasite genotyping studies indicated that current markers are not useful to indicate clinical outcome, but they clearly showed a different parasite population between Europe and South America [4].

There is a lack of epidemiological information about the frequency and clinical characteristics of the congenital infection in Colombia. In a literature survey only one study concerning pregnant women was found in a Pubmed search [5]. An additional search in non-indexed literature found 10 studies of prevalence in general population and in pregnant women from some regions of Colombia [5]. One of these studies was done in 1980 and it was representative of the general population of the country. This national screening survey determined 47% of prevalence of specific anti- *Toxoplasma* IgG in general population, indicating a high exposure to the parasite [6]. In the Quindio region a frequency of 0.6% of congenital toxoplasmosis [7], [8] and a higher frequency of ocular involvement in 36% of congenitally infected children has also been reported [9]. No information is reported in other regions of the country for congenital toxoplasmosis. Actually, there are no official control programs, despite a national guide for congenital toxoplasmosis supported by the Colombian Association of Infectious Diseases [5]. The perception of Colombian pediatricians is that only few symptomatic congenital cases are treated. Evidence of this situation was found in one survey in Cali (a city in the pacific region of Colombia) where toxoplasmosis was the second commonest cause of congenital blindness [10].

The neonatal detection of congenital toxoplasmosis was first evaluated in the region of New England in the United States [11] then in Denmark [12], [13], Poland [14], Brazil [15]–[18] and more recently in Colombia [7], [8]. Incidence rates varied from a low prevalence of 0.021% in Denmark to 0.6% live-newborn in Quindio region in Colombia. In Brazil, a large country characterized by heterogeneous socio-economic conditions and cultural and hygiene-nutritional habits, studies in newborns have reported heterogeneity in the incidence, ranging from 0.03 to 0.5% [15]–[18]. However, there have been few investigations in other South American countries that define the real magnitude of the problem. In previous investigations, confirmation of the disease was obtained by the detection of anti- *Toxoplasma* IgM antibodies in the newborn. Although this detection has been used for definitive diagnosis [11], [12] its use generally requires performing confirmatory testing. A definitive diagnosis would also be possible by the direct detection of the parasite in clinical samples, by demonstrating the persistence of anti-*Toxoplasma* IgG antibodies beyond 1 year of age [17] and/or by the detection of anti- *Toxoplasma* IgA in the first 6 months of life [14].

Considering the lack of information regarding the incidence of congenital toxoplasmosis in Colombia, we proposed a multicentric national study. The aim of this work was to determine the incidence of congenital toxoplasmosis in Colombian newborns from hospital and maternal child health services from seven different cities in five natural geographic regions (Caribbean, Central, Andean, Amazonia and the Eastern regions). The confirmatory criteria used were the persistence of anti-*Toxoplasma* IgG antibodies beyond 1 year of age or the presence of specific anti-*Toxoplasma* IgM or IgA after the 10^th^ day of life. In addition, clinical and laboratory findings for the identified infants are described.

## Methods

### Ethical aspects

Informed written consent, according to the regulation 008430 of 1993 of the Ministry of Health of Colombia, was obtained from all people that accepted to participate in the study. The University of Quindio Institutional Review Board (act number 14, 23 June 2009) approved the study. Considerations in performing this study were that obtaining umbilical cord blood samples was a non-risk procedure, the study provided an additional use for a sample that would otherwise be discarded, and that identifying infection would benefit the infected child.

### Selection of the sample and characteristics of the health institutions participating in the multicentric study

In Colombia, according to 2003–2008 UNICEF statistics and DANE (national statistics governmental institute) official reports, 92% of births occur in health institutions [19]. As part of the screening for congenital hypothyroidism, umbilical cord blood samples are taken routinely in most of regions in Colombia [20]. No other neonatal screening is performed in Colombia, excepting the search for syphilis when the mother did not have screening during pregnancy. Invitations for institutions to participate were provided during the national congress of gynecology in 2008. The only condition for participation was that the institutions which agreed to participate would instruct the personnel at the delivery room and would give information about the study to the parents. The project covered the costs of screening assays and of the laboratory assays needed for the follow up of the children. The 19 institutions that agreed to participate are listed in the Table 1, and they were from the first to the third level of referral (first level is the institution with simplest or basic level of health care and the third level is the health institution for more complex and specialized attention). Each institution was given a time to collect samples in a consecutive manner, and a limited number of samples by city was established. Collection of samples was stopped when the number of samples was reached in each city. Number of children expected to be collected in each city was: Barranquilla 3,000; Riohacha 800; Bucaramanga 3,000; Cucuta 1,000; Bogotá 5,200; Florencia 500; Armenia 1,500.

**Table 1:** Births and sample for newborn screening and referral level of participating centers.

City	Hospitals or maternal-child health care center	Referral level	Births 2009 at the hospital or center	Sample (% total births at the hospital or center)
**Armenia** (Andean)	1. Hospital Universitario San Juan de Dios	III	3,959	753 9%)
	2. Hospital La Misericordia	II	1,262	614 8%)
	3. Hospital del Sur	I	418	196 (46,8%)
**Barranquilla** (Caribbean)	4. Hospital Universidad del Norte	III	1,164	1,043 (89%)
	5. Hospital Santa Mónica	II	2,505	688 (27%)
	6. Hospital Niño Jésus	II	2,702	1,170 (43%)
**Bogota** (Center)	7. Clínica Colombia	III	4,714	544 1.5%)
	8. Hospital de Engativá	II	2,730	1,971 (72.2%)
	9. Hospital La Victoria	III	3,913	301 .6%)
	10. Hospital Simón Bolívar	III	1,771	545 0.7%)
	11. Instituto Materno Infantil	III	3,026	2,037 (67.3%)
**Bucaramanga** (Eastern)	12. Hospital de Floridablanca	II	1,700	981 7.7%)
	13. UIMIST	I	1,181	659 5.8%)
	14. Los Comuneros	III	1,068	596 6%)
	15. Local del Norte	I	1,040	560 3%)
	16. ESE Giron	I	362	202 (55%)
**Cucuta** (Eastern)	17. Hospital Erasmo Meoz	III	3,653	1,124 (30.7%)
**Florencia** Amazonian)	18. Clínica Medilaser	III	1,105	510 (46.1%)
**Riohacha** (Caribbean)	19. Hospital Nuestra Señora de los Remedios	III	2,603	801 (30.7%)

### Questionnaire and flowchart for umbilical cord blood samples

A questionnaire was required for all mothers, interrogating about age, positive IgM anti-*Toxoplasma* test or treatment for toxoplasmosis during pregnancy and SISBEN level. In Colombia, socioeconomic level of the population is determined according to the SISBEN classification, a socio-economic index [21]. The SISBEN gives an approximated measure of the magnitude of poverty, as an approximate indicator of resources or income, or as an evaluation of fulfillment of needs [21].

If mother accepted to participate in the study, blood samples from umbilical cord were collected at the moment of delivery. The personnel at the hospital's laboratory centrifuged and stored for a maximum period of 8 days at 4°C in tubes and sent to the regional laboratory.

### ELISA IgM assay

Serum from the umbilical cord blood was analyzed for anti-*Toxoplasma* IgM by a commercially available, enzyme immunocapture assay (*Toxoplasma gondii* IgM, Vircell, Grenade, Spain) according to the manufacturer's instructions. The absorbancies were measured in a microplate reader at 450 nm, with reference filter at 630 nm. Cutoff was calculated from western blot anti-*Toxoplasma* IgM negative and anti-*Toxoplasma* IgG negative samples. A receptor operating curve with 12 serums from confirmed cases (including symptomatic and asymptomatic cases) and 93 negative confirmed cases, indicated that a cutoff of index 8 detected all anti-*Toxoplasma* IgM positive cases in umbilical cord blood samples. To control for run-to-run variations, results were expressed as index, calculated by using the formula based on optical density (OD) of samples and controls: OD sample/mean OD of negative controls. The personnel at the regional laboratories received the same training to perform the technique under the same parameters. All positive samples were retested at the national reference laboratory (“Centro de Investigaciones Biomedicas”, Universidad del Quindio) again by the same ELISA assay (Vircell), a Labsystems anti-*Toxoplasma* IgM assay (Helsinki, Finland) and a western blot anti-*Toxoplasma* IgM test (LDbio, France).

### Additional criteria selection for confirmatory studies: studies for anti-*Toxoplasma* IgA and past history of diagnosis or treatment for toxoplasmosis during pregnancy

Up to 20% of infants with congenital toxoplasmosis may have negative anti-*Toxoplasma* IgM at birth [22]. Some reports established that up to half of congenitally infected cases could have anti-*Toxoplasma* IgA antibodies and that this immunoglobulin can be found in absence of specific IgM [22], [23]. It is not known if there are geographical variations about the presence of the specific IgA in absence of anti-*Toxoplasma* IgM. As an ELISA IgA was not commercially available in Colombia, we decided to use a more expensive test, the immunosorbent agglutination assay or ISAGA (Biomerieux, Lyon, France), a reference immunocapture test that use a formolized entire *Toxoplasma* antigen. Due to cost reasons, we perform the assay for anti-*Toxoplasma* IgA in a subsample of 1,613 cord blood samples that were negative in the ELISA anti-*Toxoplasma* IgM assay. Monthly, 10% of the first consecutive negative samples, from each regional laboratory, were referred to Quindio for an ISAGA IgA test. The ISAGA anti-*Toxoplasma* IgA was performed following recommendations of the manufacturer and a result ≥5 points was considered positive.

Neither obligation nor official recommendation exists in Colombia to perform serological screening for toxoplasmosis during pregnancy, but some gynecologists request the mothers do this. Particularly, in Quindio region some health insurance companies and the health bureau of the city pay for the cost for serological diagnosis of this infection in pregnant women from patients of the lowest socioeconomic levels (SISBEN 0, 1 and 2). For this reason, another criterion for postnatal follow up of the children was the mother's history of diagnosis or treatment during pregnancy for toxoplasmosis. This information was collected in the questionnaire used for this study at the moment of delivery at the hospital or maternal-child health service. Parents of these children were also invited to have their child followed clinically and serologically for congenital toxoplasmosis. Also, pediatricians participating in the study referred for confirmation, at the national reference laboratory, serum samples from cases with umbilical cord blood sample negative but that they considered presented compatible signs (hydrocephaly, cataracts or cerebral calcifications).

### Complementary assessment of mother and infant

Children who had confirmed positive results on neonatal screening for anti-*Toxoplasma* IgM or IgA or past history of treatment of toxoplasmosis during pregnancy or compatible signs were invited for diagnostic confirmation. Written, informed consent for additional assessment and follow-up was obtained from the guardians responsible for those children who tested positive. In each city there was a research assistant for the project that recalled mothers and children with criteria for follow up. Each child was examined by a neonatologist in each city, and if ophthalmological symptoms were present the child was referred to ophthalmologist. Additionally, a cerebral tomography was also indicated if physical examination was abnormal. If physical examination was normal, the children were recalled to obtain additional blood samples each month. Peripheral blood was collected from the mothers and their infants for confirmatory serological tests. During follow up mother and child were tested first for specific anti-*Toxoplasma* IgG, IgM, IgA and western blot and then only the child's sample was tested for IgG, IgM and IgA. Sera obtained from the child and mother was tested using an enzyme immune- assay for the quantitative detection of anti-*Toxoplasma* IgM by two different commercial test (Vircell, Spain and Human, Germany) and for IgA antibodies by ISAGA (Biomerieux, Lyon, France) according to the manufacturer's instructions. Samples showing optical densities higher than the cut-off provided by the manufacturer were considered positive for ELISA assays and if ≥5 points for ISAGA IgA test. Maternal and infant serum samples were also submitted to western blot to compare immunological profiles of anti-*Toxoplasma* IgG and IgM. Consequent to any alteration identified during the initial clinical assessment, the children were tested for complete blood count, cerebrospinal fluid analysis and ophthalmologic assessment. All samples from mother and child were referred to the “Centro de Investigaciones Biomédicas” at the University of Quindio.

All infants identified by neonatal screening were followed up until their infection status had been established. A diagnosis of congenital toxoplasmosis was confirmed, as described by the european network on congenital toxoplasmosis [24]. Confirmation of diagnosis was made on the basis of persistence of specific IgG antibodies beyond 12 months of age or by the presence of stable titers in absence of treatment or by the presence of compatible symptoms (cerebral calcifications, retinochoroiditis or hydrocephaly) and a specific anti-*Toxoplasma* IgG and IgM positive test in mother and of anti-*Toxoplasma* IgG antibodies in child serum [24]. Children with decreasing IgG titers and who became anti-*Toxoplasma* IgG negative in absence of treatment were considered to be non-infected. Treatment was provided based on current recommendations [9] with sulphadiazine, pyrimethamine, and folinic acid.

### Geographical factors analysis

We analyzed the differences in incidence of markers for congenital toxoplasmosis between cities by comparing geographical factors such as the altitude (meters above sea), mean annual temperature of the localities in Celsius degrees and the mean annual rainfall in mm^3^. This data were obtained from the 2008 annual report of the IDEAM (official Colombian institute for hydrological and meteorological information) [25].

### Data analysis

Calculations were performed using the Epi-Info 6 (CDC, Atlanta, GA, USA, November 1993), EpiDat 3.1 (Xunta de Galicia, Spain and OPS, Spain) and SPSS versión 14 (SPSS Inc. Chicago, USA) statistical programs. Results are expressed as the mean and standard deviation for continuous variables and N (%) for categorical variables. Correlation analyses were done by Pearson's test. Chi square test was applied to evaluate the differences in the percent of markers for congenital toxoplasmosis between cities. Epi-Info was used to perform stratified- analysis (Centers for Disease Control and Prevention [http://www.cdc.gov/epiinfo/]). Values below p<0.05 were considered statistically significant.

## Results

### Characteristics of the sample and hospitals and centers

**Figure 1:**
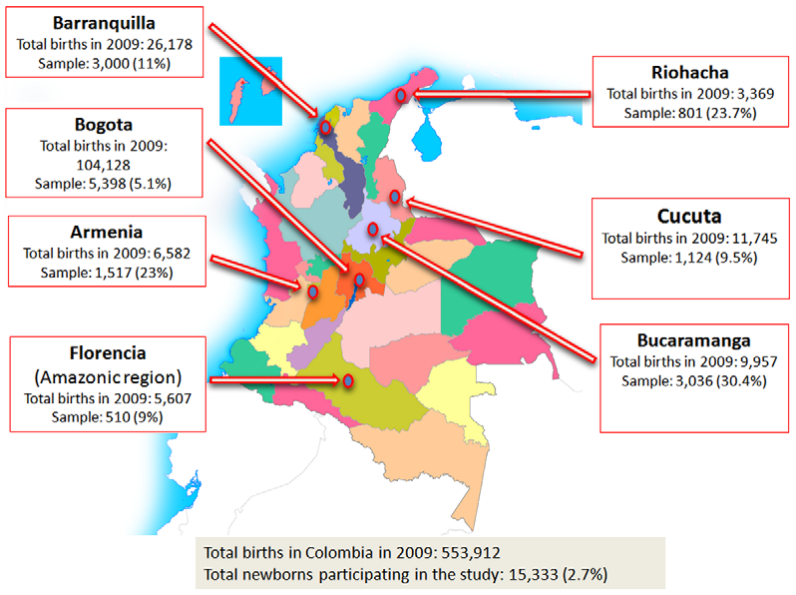
Geographical distribution and sample percent of the births. Colombian multicentric newborn screening for congenital toxoplasmosis.

The newborns studied (15,333) accounts for 2.7% of the 553,912 live newborns delivered in 2009 in Colombia and for the 9.1% of births in the seven cities included in this study, N: 167,566 (Figure 1). The size of the sample related to the total of births during 2009, by each hospital or center, and the referral level is depicted in Table 1. Only 300 samples in Barranquilla (Hospital Universidad del Norte) and 400 in Bogota (Hospital Engativa) were collected during the first five months of 2010. The 47% (n: 9) of the participating hospitals were from the third level, 31% (n: 6) from the second level and 21% (n: 4) from the first level of referral. The 96.5% of the newborns were from hospitals and maternal- child services that received patients of the levels 0 to 2, which are the lowest levels of SISBEN (Table S1). Only 3.5% (n: 544) of the sample was from one hospital with a majority of population with levels of SISBEN greater than 3 (Clinica Colombia in Bogota).

### Frequency of anti-*Toxoplasma* IgM and IgA antibodies in umbilical cord blood samples

In the regional laboratories 118 umbilical cord blood samples were positive for anti-*Toxoplasma* IgM ELISA assay, but then only 61 (51.7%) were confirmed by the western blot and retesting at the reference laboratory. Consequently the prevalence for confirmed specific IgM was of 0.39%. Nine samples of 1,613 tested were positives for ISAGA IgA assay (0.5%). Results by hospital or maternal child health care center and city are shown in Table 2. The rate of confirmed positive anti-*Toxoplasma* IgM test in cord blood samples by grouping hospitals and centers by city was: Bogota 0.2% (Central region); Bucaramanga 0.23% and Cucuta 0% (Eastern region), Barranquilla 0.06% and Riohacha 0% (Caribbean region); Armenia 2.0% (Andean region) and Florencia 1.8% (Amazonia). For specific IgA anti-*Toxoplasma* we found: Bogotá 0.39%; Bucaramanga 0.78%; Barranquilla 1.2%, Armenia 0%, Cucuta 0%, Florencia 0% and Riohacha 0%.

**Table 2:** Frequency of markers for congenital toxoplasmosis in hospitals and maternal child care centers in 7 cities.

City	Toxoplasmosis history during pregnancy	IgM umbilical cord blood	IgA umbilical cord blood	Hospitals or maternal child centers
Armenia	27/753 (3.5%)	6/753 (0.79%)	0/75 (0%)	1. Hospital San Juan de Dios
	35/568 (6.1%)	19/568 (3.3%)	0/57 (0%)	2. Hospital La Misericordia
	5/196 (2.5%)	6/196 (3.0%)	0/19 (0%)	3. Hospital del Sur
**Total in Armenia**	**64/1517 (4.2% IC95% 3.4–5.3)**	**31/1517 (2.0% IC95%1.3–2.8)**	**0/151 (0% IC95% 0–2.4)**	**Total markers: 6.2%**
Barranquilla	6/1043 (0.5%)	1/1043 (0.09%)	0/104 (0%)	4. H. Universidad del Norte
	7/688 (1.0%)	0/688 (0%)	1/95 (1.0%)	5. Hospital Santa Mónica
	4/1170 (0.3%)	1/1170 (0.8%)	3/117 (2.5%)	6. Hospital Niño Jésus
**Total in Barranquilla**	**17/2901 (0.58% IC95% 0.5–1.3)**	**2/2901 (0.06% IC95% 0–.2)**	**4/316 (1.2% IC95% 0.3–3.3)**	7. **Total markers:1.8%**
Bogota	5/544 (0.9%)	4/544 (0.73%)	0/54 (0%)	8. Clínica Colombia
	15/1971 (0.76%)	2/1971 (0.1%)	1/191 (0.52%)	9. Engativa
	0/301 (0%)	0/301 (0%)	0/30 (0%)	10. La Victoria
	2/545 (0.36%)	1/545 (0.18%)	0/28 (0%)	11. Simon Bolivar
	14/2037 (0.68%)	5/2037 (0.24%)	1/203 (0.49%)	12. Instituto Materno Infantil
**Total in Bogota**	**36/5398 (0.66% IC95% 0.4–0.8)**	**12/5398 (0.2% IC95% 0.08–0.3)**	**2/506 (0.39% IC95%0.04–1.4)**	13. **Total markers: 1.2%**
Bucaramanga	2/1.019 (0.19%)	1/1.019 (0.09%)	1/141 (0.7%)	14. Hospital de Floridablanca
	0/659 (0%)	2/659 (0.3%)	1/76 (1.3%)	15. UIMIST
	7/596 (1.1%)	1/596 (0.16%)	0/69 (0%)	16. Los Comuneros
	0/560 (0%)	3/560 (0.53%)	1/66 (1.5%)	17. Local del Norte
	1/202 (0.49%)	0/202 (0%)	0/30 (0%)	18. ESE Giron
**Total in Bucaramanga**	**10/3036 (0.32% IC95%0.1–0.5)**	**7/3036 (0.23% IC95%0.04–0.4)**	**3/382 (0.78% IC95%0.1–2.2)**	19. **Total markers: 1.3%**
**Cucuta**	**6/1124 (0.53% IC95% 0.06–1)**	**0/1124 (0% IC95% 0–0.3)**	**0/110 (0% IC95% 0–3.2)**	20. Hospital Erasmo Meoz; **Total markers: 0.5%**
**Florencia**	**4/510 (0.78% IC95% 0.2–1.9)**	**9/510 (1.8% IC95% 0.5–3)**	**0/56 (0% IC95% 0–6.3)**	21. Clínica Medilaser; **Total markers: 3.1%**
**Riohacha**	**6/801 (0.74% IC95% 0.09–1.4)**	**0/801 (0% IC95% 0–0.4)**	**0/92 (0% IC95% 0–3.9)**	22. Nuestra Señora de los Remedios; **Total markers: 0.7%**

### Frequency of history of toxoplasmosis during pregnancy and children with compatible signs

A history of diagnosis for toxoplasmosis during pregnancy was found in 143 questionnaires and from these mothers, 82 received antibiotics (79 spiramycin, and three pyrimethamine- sulfadoxine combination). From these mothers only two children whose mothers were treated (one with pyrimethamine-sulfadoxine during two months and one with spiramycin during three months) had anti-*Toxoplasma* IgM test positive in umbilical cord blood, however in the follow up both children become negative.

Five samples of serum from children who were negative at the umbilical cord blood tests (four from Bogota and one from Barranquilla) were referred because the newborn presented clinical signs compatible with congenital toxoplasmosis (hydrocephaly, cerebral calcifications or cataracts). In these cases mothers did not have history of toxoplasmosis during pregnancy and they did not received treatment during pregnancy.

### Frequency of confirmed and symptomatic congenital infection

**Figure 2:**
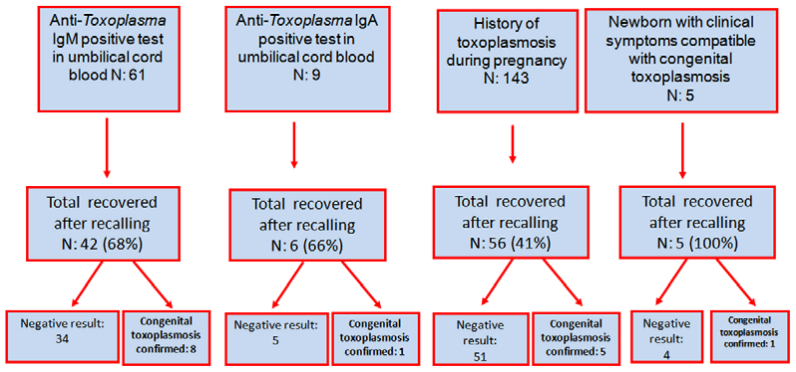
Flowchart and results of the postnatal confirmatory tests at the follow up.

We found and followed 109 of the 218 children that had some of the criteria for postnatal confirmatory tests (Figure 2). We confirmed a congenital toxoplasmosis infection in 15 children: 7 were symptomatic, and three of them died before the first month of life. Eight infected children were identified by a positive specific IgM test in umbilical cord blood sample, one by a positive specific IgA test in umbilical cord blood, five by the past history of treatment during pregnancy and one by compatible symptoms. A summary of the clinical characteristics, criteria for recruitment and for diagnosis is showed in Table 3.

**Table 3:** Clinical and serological characteristics in 15 children with confirmed congenital *Toxoplasma* infection.

Initials-(City)	Signs or symptoms?	Diagnostic criteria after day 10 of life	Index IgM umbilical cord blood sample	Criteria for following
1. SDG (Armenia)	Asymptomatic	IgG, IgM and IgA positives in child	**9,8**	IgM+umbilical cord
2. JE (Armenia)	Asymptomatic	IgG and IgM positives in child	**12,3**	IgM+umbilical cord
3. SR (Armenia)	Asymptomatic	IgG, IgM and IgA positives in child	**19,9**	IgM+umbilical cord
4. AM (Bogotá)	Choriorretinitis and cerebral calcifications	IgG and IgA positives in child in month five of life	0	Compatible signs
5. SM (Bogotá)	Splenomegaly	Stable titers IgG, IgA+	0	IgM+mother
6. PAC (Florencia)	Preterm, calcifications, death after 3 weeks of life	IgG and IgM positives in child	**13,2**	IgM+umbilical cord
7. MH (Bucaramanga)	Neurological soft signs, cerebral tomography normal	IgG, IgM and IgA positives in child	**12**	IgM+umbilical cord
8. YB (Barranquilla	Preterm, choriorretinitis, cerebral calcifications	IgG e IgM positives in child	0,6	IgA+umbilical cord
9. LP (Barranquilla)	Asymptomatic	IgG, IgM and IgA positives in child	**11**	IgM+umbilical cord
10. ZCs (Bogota)	Asymptomatic	IgG positive after month 12	2,2	IgM+mother and 6 months of **treatment with spyramicin during pregnancy**
11. AM (Bogota)	Asymptomatic	IgG positive after month 12	**8,8**	IgM+mother and 2 months of **treatment with spyramicin during pregnancy**
12. YR (Bogota	Asymptomatic	IgG positive after first year of life, IgG stable titers	**9,8**	IgM+umbilical cord
13. OA (Barranquilla)	Hydrocephaly, cerebral calcifications, choriorretinitis, death at first week of life	Compatible symptoms, mother was IgM positive in pregnancy	4,1	IgM+mother
14. ANS (Florencia)	Respiratory distress syndrome, death after three weeks of life	Compatible symptoms, mother was IgM and IgA positives	**10,4**	IgM+umbilical cord
15. JPM (Armenia)	Asymptomatic	PCR amniotic fluid positive, IgG stable titers	2,2	IgM+mother and **treatment during pregnancy**

In newborns of mothers with history of diagnosis or treatment for toxoplasmosis during pregnancy, 56 children could be followed and five were confirmed as congenitally infected (8.9%). During pregnancy, two mothers of the confirmed congenitally infected children were treated with spiramycin and one with pyrimethamine- sulfadoxine combination, and children were asymptomatic. The other two children did not received prenatal treatment, one of them died during the first week of life and another had splenomegaly.

One child who was referred because he was presenting compatible signs for congenital toxoplasmosis, had specific anti-*Toxoplasma* IgA test positive in peripheral blood five months after birth, that confirmed congenital toxoplasmosis. The child was not treated prenatally and had choriorretinitis.

In the postnatal confirmatory samples, seven children had a specific anti-*Toxoplasma* IgM positive test and six children had a specific anti-*Toxoplasma* IgA positive test. Between the seven children with specific IgM positive, four of them also had specific IgA. Two children with specific IgA positive test did not have specific IgM.

Three of 15 children (20%) with confirmed congenital toxoplasmosis died during the first month of life: one in Barranquilla and other two in Florencia. The mothers of these three children had positive tests for anti-*Toxoplasma* IgG and IgM. In the three cases there was no suspicion of toxoplasmosis by pediatricians that attended the cases. Cerebral tomography of one of the cases in Florencia is shown on Figure S1. In this particular case, the cell culture from cerebral spinal fluid showed growth of *Toxoplasma* tachyzoites, confirmed by direct immunofluorescence assay. In contrast, three prenatally treated children were asymptomatic. The lethality rate in 12 prenatally untreated children for this newborn congenital toxoplasmosis screening program was of 25%.

### Geographical factors influencing frequency of serological markers for congenital toxoplasmosis

Incidence of specific anti-*Toxoplasma* IgM in umbilical blood cord (p = .000) and of the percent of mothers with history of toxoplasmosis during pregnancy (p: 0.000), were significantly different between cities, but not the incidence of specific IgA (p: 0.22). As the study involved different type of institutions, we analyzed the influence of socioeconomics classification, referral level and geographical factors on the incidence of markers at birth for congenital toxoplasmosis: incidence of anti-*Toxoplasma* IgM or IgA in umbilical cord or frequency of history of toxoplasmosis during pregnancy (Table 4).

**Table 4:** Rate of markers for congenital toxoplasmosis and geographical characteristics.

City (size of the sample, number of hospitals and centers)	% markers for congenital toxoplasmosis	Altitude above sea -meters-	Average temperature °C	Mean annual precipitation mm^3^
Florencia(n: 510, one hospital)	3.1	242	30	3,840
Armenia(n: 1.563, two hospitals, one center)	6.2	1,551	26	2,500
Barranquilla(n: 2.901, three hospitals)	1.8	0	27	821
Bogota(n: 5.398, five hospitals)	1.2	2,642	12	995
Bucaramanga(n: 3.036, four hospitals, one center)	1.3	960	24	1,279
Cucuta(n: 1.124; one hospital)	0.5	320	28	806
Riohacha(n: 801; one hospital)	0.7	52	28	48

Pearson correlation test showed that neither referral level nor socioeconomics index was correlated with the incidence of markers for congenital toxoplasmosis. Concerning the geographical factors, altitude or average temperature of the city were not correlated with the incidence of markers, but the mean annual rain fall was significantly correlated to differences in the frequency of serological markers for congenital toxoplasmosis in each city (Table 5).

**Table 5:** Pearson test for correlations at the Colombian multicentric newborn screening for congenital toxoplasmosis.

		Referral level of the center	Altitudes (meters above sea)	Average temperature of the city °C	Mean rainfall mm^3^	SISBEN level ≥3	Mean age years of the mother	Incidence markers (IgM and IgA umbilical cord and past history during pregnancy)
**Referall level of the center**	Pearson Correlation	1	0.128	−0.219	−0.100	0.282	**0.575**(*)	−0.257
	Sig. (2-tailed)		0.601	0.368	0.685	0.273	**0.016**	0.287
	N	19	19	19	19	17	17	19
**Altitude**	Pearson Correlation	0.128	1	**−0.907**(**)	0.014	**0.568**(*)	0.050	−0.007
	Sig. (2-tailed)	0.601		**0.000**	0.955	**0.017**	0.848	0.978
	N	19	19	19	19	17	17	19
**Average Temperature**	Pearson Correlation	−0.219	**−0.907**(**)	1	0.307	**−0.694**(**)	−0.305	0.342
	Sig. (2-tailed)	0.368	**0.000**		0.201	**0.002**	0.234	0.152
	N	19	19	19	19	17	17	19
**Mean rainfall mm^3^**	Pearson Correlation	−0.100	0.014	0.307	1	−0.110	−0.267	**0.612**(**)
	Sig. (2-tailed)	0.685	0.955	0.201		0.675	0.300	**0.005**
	N	19	19	19	19	17	17	19
**SISBEN level ≥3**	Pearson Correlation	0.282	0.568(*)	**−0.694**(**)	−0.110	1	**0.785**(**)	−0.185
	Sig. (2-tailed)	0.273	0.017	**0.002**	0.675		**0.000**	0.477
	N	17	17	17	17	17	17	17
**Mean age in years of the mother**	Pearson Correlation	**0.575**(*)	0.050	−0.305	−0.267	**0.785**(**)	1	−0.367
	Sig. (2-tailed)	**0.016**	0.848	0.234	0.300	**0.000**		0.148
	N	17	17	17	17	17	17	17
**Incidence of markers**	Pearson Correlation	−0.257	−0.007	0.342	**0.612**(**)	−0.185	−0.367	1
	Sig. (2-tailed)	0.287	0.978	0.152	**0.005**	0.477	0.148	
	N	19	19	19	19	17	17	19

*Correlation is significant at the 0.05 level (2-tailed).

**Correlation is significant at the 0.01 level (2-tailed).

## Discussion

This is the first multicentric study in Colombia of congenital toxoplasmosis incidence. As the selection of the sample was on the basis of voluntary acceptation to participate, the sample is representative only for the centers involved in the study. However, they should provide representative data for the incidence in the city where they were obtained. Incidence in toxoplasmosis could differ by the socioeconomics, age or geographical factors [26] but in present study these variables not determined the differences in incidence of congenital toxoplasmosis between cities. Thus, the incidence of markers indicates a similar situation to the described in Brazil [27]. The findings suggest epidemiological differences in infection in distinct localities. Our methods are not influenced by inclusion of cases or more sensitive methods because cases were recruited by the same criteria (every child born at each hospital or center during the period of sample collection), test were free of costs for parents, no refusal was recorded from their parents, laboratories performed the same assays, personnel received the same training and all positive samples were confirmed at the referral center by the same methodology.

This study found three children that died before first month of life as a consequence of the congenital toxoplasmosis. In previous reports, of newborn screening, mortality cases between congenitally infected children were also described: one between 19 newborns in the study in Poland [14] and one case between 20 newborns in Porto Alegro's study [15]. Although our rate of mortality seems higher, it is difficult to conclude about the real magnitude of the problem due to the high number of children loss of follow up. Future studies should focus in to obtaining a complete follow up of all of the cases, but it is clear that congenital toxoplasmosis is an important cause of children early mortality in Colombia.

We did an aggregation of the three markers because each one independently indicates the risk for congenital toxoplasmosis. Thus, anti-*Toxoplasma* IgA marker was obtained in the anti-*Toxoplasma* IgM negative sample and history of toxoplasmosis during pregnancy identified children without specific IgM or IgA in umbilical cord sample. For these reasons we considered that complete evaluation of the risk for congenital toxoplasmosis cannot be attained without considering other factors besides the presence of specific IgM in umbilical cord blood. This seems critical for cities as Barranquilla and Bucaramanga which had very low incidence of IgM but the highest incidence of positive IgA. Therefore it is not possible to say that congenital toxoplasmosis does not exist in these cities without considering the other markers. Furthermore, in cities such as Cucuta and Riohacha all three markers were very low, being consistent with this assumption.

Previous studies in others countries have targeted the frequency of IgM or IgA in umbilical cord as markers for congenital infection, however as some mothers received treatment and this can influence the presence of serological markers at birth in congenitally infected children [28], we included into the analysis the history of diagnosis and treatment for toxoplasmosis during pregnancy. The results of incidence of anti-*Toxoplasma* IgM in umbilical cord blood samples and of the percentage of mothers with history of diagnosis or treatment during pregnancy were correlated, so cities with hospitals and centers with high incidence of mothers with history of toxoplasmosis during pregnancy also had high incidence of IgM in umbilical cord blood sample. In contrast, the incidence of IgA was not correlated with these markers. Anti- *Toxoplasma* IgA positive test in newborns was high in cities where anti-*Toxoplasma* IgM in cord blood was low. In cities where hospitals and centers had a high incidence of IgM, the anti-*Toxoplasma* IgA in umbilical cord blood it was not found exclusively.

Anti-*Toxoplasma* IgA can be found alone without anti-*Toxoplasma* IgM. In present study, two children with confirmed congenital infection had IgA as the only diagnostic marker of congenital infection after day ten of life. A possible explanation for the differences in the incidence of anti-*Toxoplasma* IgA between cities are the consumption of the IgM production before birth in infections acquired early during pregnancy [13], [23], [28]. Therefore in these cities the pattern of acquisition can differ and infections can occur earlier than in other cities. Other alternative explanation is genetic differences in the population to produce IgA or differences in the *Toxoplasma* strains between cities. It should be also noted that IgM is more often negative in the symptomatic infants, because of the earlier infection in fetal life. In asymptomatic patients, the frequency of positive IgM is much higher [13], [23], [28].

The factors associated to the incidence of each positive marker independently or altogether did not correlate to the level of SISBEN or to the level of referral of the center. Looking for geographical links between cities with hospitals and centers with high or low incidence of markers for congenital toxoplasmosis, we analyzed the altitude, the mean temperature and the mean annual rainfall. We found that mean annual rainfall was strongly related to the level of incidence of markers for congenital toxoplasmosis. The association with this geographical parameter is not surprising; the first studies about survival of sporulated *Toxoplasma gondii* oocysts in water and soil indicated that humidity is a critical parameter for their infectivity [29], [30]. In France, a study about the incidence risk in cats was related to mean precipitation, explaining both the spatial and temporal variability in risk: local conditions explained differences between the study sites and incidence risk increased during rainy years [31].

One advantage of the present study design is that the occurrence of congenital toxoplasmosis in infants identified by neonatal screening was confirmed using a criteria that is considered to be a true indicator of the presence or absence of the disease [24], but that has been applied in only few previous studies. The confirmation of the results in the umbilical cord sample indicated that the test we use was few specific in a similar manner to the reported with the fluorescence based assay in Brazil. In present study, 51.6% of the initially positive neonatal screening results were false-positive. Other investigators, using IgM fluorometric assay reported 61.5% [17], 78.6% [32] and 91% [15] false-positive results. A high proportion of false-positive results are generally accepted for screening tests, especially in the case of populations with low incidence of the disease.

n summary, the first Colombian multicentric study identified symptomatic and asymptomatic congenital infected children and a high lethality rate in prenatally untreated children (25%) in hospitals and centers from different level of referral in five of seven cities participating at the study. This program identified nine children otherwise undiagnosed (no previous history in mother or no clinical suspicion). For the first time a significant correlation was identified between mean rainfall at the city and the incidence of markers for congenital infection. In Colombia, each city or region should define the necessity of newborn screening for congenital toxoplasmosis according to local studies.

## Supporting Information

Figure S1 Cerebral tomography of one confirmed case of congenital toxoplasmosis at the city of Florencia (Amazonian region).
(DOCX)

Table S1 Mean age ± standard deviation (SD) and percent for each level of SISBEN socioeconomic classification in population from hospitals and maternal- child health centers participating in the study.
(DOCX)
