# Supplementary material for: First Colombian Multicentric Newborn Screening for Congenital Toxoplasmosis
Source: PLoS Negl Trop Dis. 2011 May 31;5(5):e1195. doi: 10.1371/journal.pntd.0001195 (PMC3104965; doi:10.1371/journal.pntd.0001195)

**Figure S1.** Cerebral tomography of one confirmed case of congenital toxoplasmosis at the city of Florencia (Amazonian region). The child born in 30 July, 2009 with an estimated gestational age by Capurro scale of 32 weeks. Mother had one serology for toxoplasmosis at the beginning of the pregnancy that gave a negative result without additional controls. The child presented a respiratory distress syndrome and died in September 18, 2009. Mother has 348 UI/ml and the child 294 UI/ml of IgG anti-*Toxoplasma* by ELISA assay and an anti-*Toxoplasma* IgM ELISA test result in the mother’s serum of 2.9 and in the child’s serum of 2.1 (cutoff: 1.08) in 28 August of 2009.


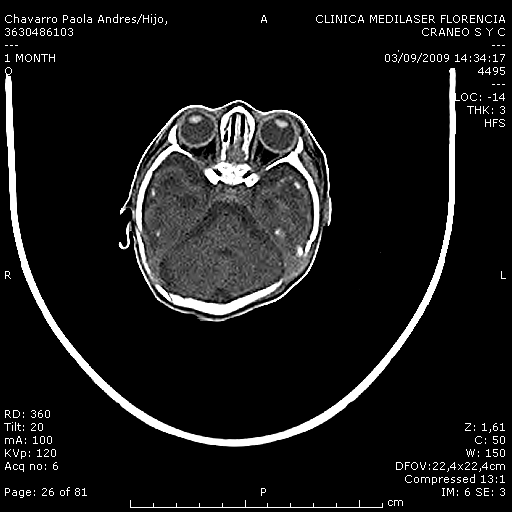

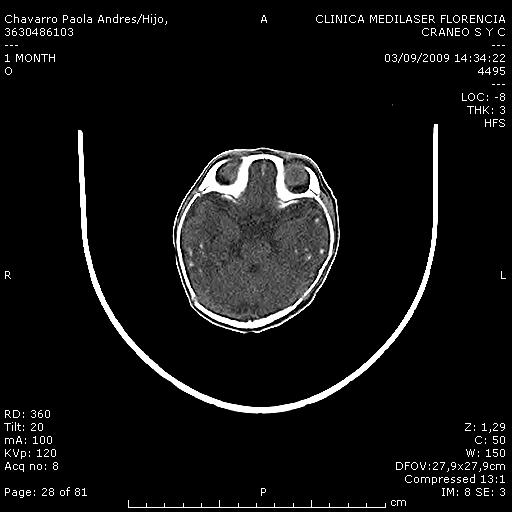


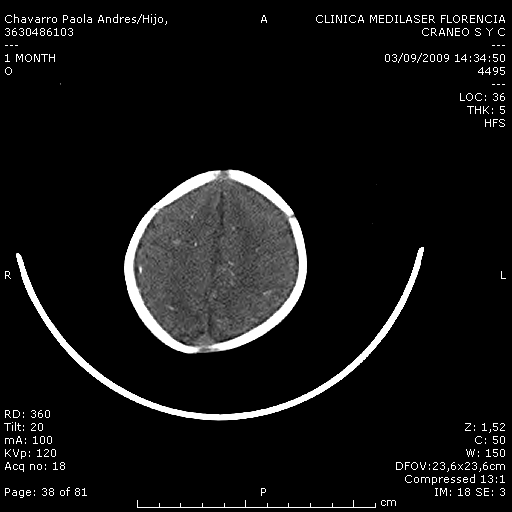

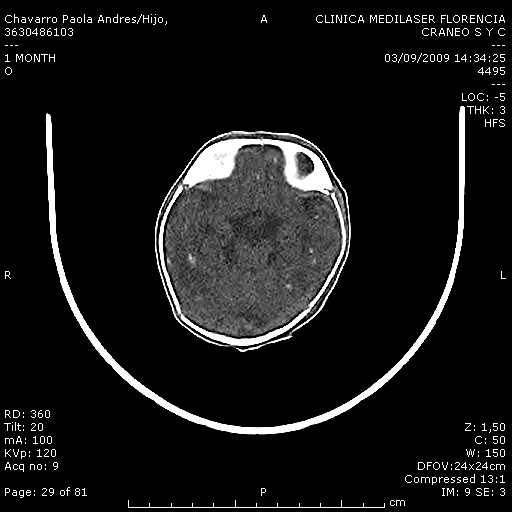

Supplement: Figure S1 — Cerebral tomography of one confirmed case of congenital toxoplasmosis at the city of Florencia (Amazonian region). (DOCX) [file pntd.0001195.s001.docx]
