# Supplementary material for: First Colombian Multicentric Newborn Screening for Congenital Toxoplasmosis
Source: PLoS Negl Trop Dis. 2011 May 31;5(5):e1195. doi: 10.1371/journal.pntd.0001195 (PMC3104965; doi:10.1371/journal.pntd.0001195)
Supplement: Table S1 — Mean age ± standard deviation (SD) and percent for each level of SISBEN socioeconomic classification in population from hospitals and maternal- child health centers participating in the study. (DOCX) [file pntd.0001195.s002.docx]

**Table S1.** Mean age ± standard deviation (SD) and percent for each level of SISBEN socioeconomic classification in population from hospitals and maternal- child health centers participating in the study. The information is based on 9,939 complete questionnaires of the 15,333 newborns. Hospital Simon Bolivar and La Victoria in Bogota did not reported this information. 0 is the lowest level and 6 the highest.

| **Hospital or maternal child health service (n: number of questionnaires with information)** | **Mean age of patients± SD** | **SISBEN 0** | **SISBEN 1** | **SISBEN 2** | **SISBEN 3** | **SISBEN 4** | **SISBEN 5** | **SISBEN 6** |
| --- | --- | --- | --- | --- | --- | --- | --- | --- |
| 1. Hospital San Juan de Dios (n: 224) | 23.4±6.6 | 0 | 58 | 36.6 | 4.0 | 0.9 | 0 | 0.4 |
| 1. Hospital La Misericordia (n: 385) | 22.5±6.2 | 0 | 84.2 | 15.3 | 0.5 | 0 | 0 | 0 |
| 1. Hospital del Sur (n: 38) | 22.4±5.1 | 0 | 71 | 23.7 | 5.3 | 0 | 0 | 0 |
| 1. Hospital Universidad del Norte (n: 1.031) | 25.5±5.6 | 0 | 46.3 | 46 | 7.5 | 0 | 0.3 | 0 |
| 1. Hospital Santa Mónica (n: 660) | 26.2±5.7 | 0 | 45.5 | 37.1 | 13.5 | 3.2 | 0.8 | 0 |
| 1. Hospital Niño Jésus (n: 1.142) | 23.3±5.8 | 0 | 80.8 | 18 | 1.1 | 0 | 0 | 0 |
| 1. Clínica Colombia (n: 383) | 28.1±6 | 0 | 2.3 | 24.5 | 56.9 | 14.4 | 1.3 | 0.5 |
| 1. Engativá (n: 1.046) | 24±6 | 0.7 | 26.6 | 44 | 28.6 | 0.2 | 0 | 0 |
| 1. IMI (n: 1.149) | 24±6 | 0.2 | 43.4 | 41.1 | 15.2 | 0.1 | 0 | 0 |
| 1. Hospital de Floridablanca (n: 668) | 23±6.0 | 0.1 | 60 | 39.7 | 0 | 0 | 0 | 0 |
| 1. ESE Giron (n: 127) | 22±4.7 | 0.8 | 89 | 10.2 | 0 | 0 | 0 | 0 |
| 1. UIMIST (n: 491) | 23±6.5 | 0.8 | 69.7 | 29.5 | 0 | 0 | 0 | 0 |
| 1. Los Comuneros (n: 319) | 24±6 | 0 | 36.7 | 58.3 | 0 | 5 | 0 | 0 |
| 1. Local del Norte (n: 383) | 23±5.9 | 1.0 | 75.2 | 23.8 | 0 | 0 | 0 | 0 |
| 1. Hospital Erasmo Meoz (n: 563) | 23.8±6.5 | 0.9 | 74.2 | 23.4 | 1.1 | 6.4 | 0 | 0 |
| 1. Clínica Medilaser (n: 530) | 24.6±6.6 | 0.8 | 57.2 | 32.1 | 9.8 | 0.2 | 0 | 0 |
| 1. H. Nuestra Señora de los Remedios (n: 800) | 24.5±6.9 | 0 | 95.1 | 4.9 | 0 | 0 | 0 | 0 |
